# Intratumoural-infiltrating CD4 + and FOXP3 + T cells as strong positive predictive markers for the prognosis of resectable colorectal cancer

**DOI:** 10.1038/s41416-019-0559-6

**Published:** 2019-09-06

**Authors:** Taichi Kuwahara, Shoichi Hazama, Nobuaki Suzuki, Shin Yoshida, Shinobu Tomochika, Yuki Nakagami, Hiroto Matsui, Yoshitaro Shindo, Shinsuke Kanekiyo, Yukio Tokumitsu, Michihisa Iida, Ryouichi Tsunedomi, Shigeru Takeda, Shigefumi Yoshino, Naoko Okayama, Yutaka Suehiro, Takahiro Yamasaki, Tomonobu Fujita, Yutaka Kawakami, Tomio Ueno, Hiroaki Nagano

**Affiliations:** 1 Department of Gastroenterological, Breast and Endocrine Surgery, Yamaguchi University Graduate School of Medicine, Ube, Yamaguchi 755-8505 Japan; 2 Department of Translational Research and Developmental Therapeutics against Cancer, Yamaguchi University Faculty of Medicine, Ube, Yamaguchi 755-8505 Japan; 3 Oncology Center, Yamaguchi University Hospital, Ube, Yamaguchi 755-8505 Japan; 4 Division of Laboratory, Yamaguchi University Hospital, Ube, Yamaguchi 755-8505 Japan; 5 Department of Oncology and Laboratory Medicine, Yamaguchi University Graduate School of Medicine, Ube, Yamaguchi 755-8505 Japan; 6 Division of Cellular Signaling, Institute for Advanced Medical Research, Keio University School of Medicine, Shinjuku, Tokyo 160-8582 Japan; 7 Department of Digestive Surgery, Kawasaki Medical University, Kurashiki, 701-0192 Okayama Japan

**Keywords:** Colon cancer, Tumour immunology

## Abstract

**Background:**

CD3 +  and CD8 + T-cell infiltration were reported as positive predictive markers of survival in colorectal cancer (CRC) patients. Here, we demonstrate the prognostic significance of CD4 + and FOXP3 + T-cell densities in CRC.

**Methods:**

We quantified the intratumoural densities of CD3 + , CD8 + , CD4 +  and FOXP3 + T cells by immunohistochemistry and digital pathology in 342 CRC patients who underwent curative resection. Microsatellite instability was also assessed in 322 specimens. Patient demographics, clinicopathological features and survival rates were analysed.

**Results:**

High CD3 + , CD4 +  and FOXP3 + T-cell densities were associated with improved relapse-free survival (RFS); high CD8 + , CD4 +  and FOXP3 + T-cell densities were associated with improved disease-specific survival (DSS). Patients with low CD4 + and low FOXP3 + T-cell densities exhibited extremely poor prognoses. T stage, vascular/lymphatic invasion and CD4 + T-cell density were independent prognostic indicators for DSS. The distributions of CD4 + and FOXP3 + T-cell densities were not significantly different between the high microsatellite instability group and other groups, in contrast to those of CD3 + and CD8 + T-cell densities.

**Conclusions:**

Intratumoural CD4 + T-cell density and combined CD4 + and FOXP3 + T-cell densities were stronger prognostic indicators than other clinicopathological features. These results may facilitate the establishment of novel prognostic factors and therapeutic strategies for CRC.

## Background

Colorectal cancer (CRC) is the third most common cancer and the fourth leading cause of cancer-related deaths worldwide; it results in more than 500,000 deaths annually.^1,2^ Despite recent improvements in surgical treatments and the development of chemotherapy, deadly disease recurrence occurs in 20–25% of patients, and the effectiveness of treatments remains unsatisfactory.^3^

The Union for International Cancer Control (UICC) tumour, node and metastases (TNM) classification has been shown to be valuable for estimating patient prognosis.^4,5^ However, this approach alone is insufficient as a prognostic predictor because clinical outcomes can differ between patients at the same histologic tumour stage.^6^ Adjuvant chemotherapy has been widely recommended as standard treatment for stage III CRC patients since the early 1990s;^7^ currently, high-risk stage II patients are also treated with adjuvant chemotherapy.^8^ There are many kinds of chemotherapy regimens, such as fluoropyrimidines alone or in combination with oxaliplatin, the intensities of which differ according to the stage of the disease.^9^ Hence, the identification of novel predictive biomarkers is needed to categorise and identify patients who could receive the greatest benefit from adjuvant chemotherapy.^9–11^

Mutation status and tumour gene expression-based classification methods only have a moderate predictive accuracy and limited clinical usefulness for predicting the risk of recurrence.^12–14^ Recently, many reports have demonstrated the favourable prognostic impact of in situ immune cell infiltration in tumours.^15–17^ Tumour-infiltrating lymphocytes (TILs), especially CD3 + and CD8 + T cells,^17,18^ may act as indicators of the host immune response to the tumour, and may represent a strong independent positive predictor of relapse and overall survival. In addition, a scoring system called Immunoscore, which summarises the densities of CD3 + and CD8 + T-cell effectors within the tumour and its invasive margins, has been shown to be useful in predicting the clinical outcome of patients with CRC.^19,20^

In contrast, CD4 + T cells are less well defined as a prognostic marker in CRC, and few studies have reported a significant association between CD4 + T-cell density and survival.^21,22^ In addition, currently, forkhead box P3 (FOXP3) + cells are generally considered to be immunosuppressive in many types of cancers,^23,24^ with the exception of CRC, in which investigations of FOXP3 + cell infiltration have shown conflicting results.^25,26^

In this study, we investigated not only CD3 + and CD8 + but also CD4 + and FOXP3 + T cells from resected specimens of primary CRC patients who had undergone curative resections to clarify their prognostic value. Here, to our knowledge, we demonstrated for the first time that intratumoural CD4 + and FOXP3 + cell infiltration may be the most meaningful predictive factor in CRC patients.

## Methods

### Patients and tissue samples

Cancer tissue specimens were obtained from a consecutive series of 342 patients who underwent curative resections for CRC at the Department of Gastroenterological, Breast and Endocrine Surgery, Yamaguchi University Graduate School of Medicine, Japan, from 1993 to 2012. Patients with a tumour below the peritoneal reflection were excluded from the study to exclude lower rectal cancer patients, whose preoperative treatment as neoadjuvant chemo-(radiation) therapy has been differ from colon cancer upper the peritoneal reflection,^27^ and those who died from other diseases or stopped follow-up within 5 years after resection were excluded to determine the 5-year disease-specific survival (DSS) and relapse-free survival (RFS). The patient demographics and baseline characteristics are listed in Table 1. Adjuvant chemotherapy was performed mainly for stage III and high-risk stage II CRC patients. The percentages of patients who received adjuvant therapy were 11, 64 and 87% for Stage I, II and III CRC, respectively (Table S1). Ethical, legal and social implications were approved by the Ethics Committee of Yamaguchi University Hospital (H17-83 and H23-135). All samples were obtained with the patients’ informed consent.

**Table 1:** Demographics and baseline characteristics (*n* = 342)

Characteristic	No. (%)
*Age, years (range)*	
Median	69
Range	27–95
< 65	124 (36)
*Sex*	
Male	179 (52)
Female	163 (48)
*Primary tumour location*	
Ascending colon	94 (27)
Transverse colon	36 (11)
Descending and sigmoid colon	135 (39)
Upper rectum*	77 (23)
*Disease stage at diagnosis*	
I	88 (26)
II	142 (41)
III	112 (33)
*T stage*	
T1	54 (16)
T2	48 (14)
T3	211 (62)
T4	29 (8)
*N stage*	
N0	230 (67)
N+	112 (33)
*Histologic grade*	
Well differentiated	92 (27)
Moderately differentiated	223 (65)
Poorly differentiated	16 (5)
Mucinous/undifferentiated	11 (3)
*Vascular emboli and lymphatic invasion*	
Present	172 (51)
Absent	170 (49)
Perforation	
Present**	8 (2)
Absent	334 (98)

*Above the peritoneal reflection. **Four patients out of eight cases with perforation experienced relapses within 3 years

### Immunohistochemistry and the analysis of TILs

Immunohistochemistry (IHC) was performed as previously described.^17,28^ Briefly, after the pathological assessment of haematoxylin and eosin-stained slides of the surgical specimens, 4-μm sections were made from formalin-fixed, paraffin-embedded specimens. IHC staining was carried out automatically using the Ventana Discovery XT staining system (Ventana, Tucson, AZ, USA). To identify the types of TILs, the following four antibodies were used: anti-CD3 (rabbit monoclonal, 518110079 (CD3); Ventana), anti-CD8 (mouse monoclonal, 1:50, IR623; Dako, Foster City, CA, USA), anti-CD4, (mouse monoclonal, 518108816 (CD4); Ventana) and anti-FOXP3 (mouse monoclonal, 1:100, ab20034; Abcam, Cambridge, MA, USA). As an isotype control, anti-mouse IgG1 antibody (1:100, ab91353; Abcam) was also used.

**Fig. 1:**
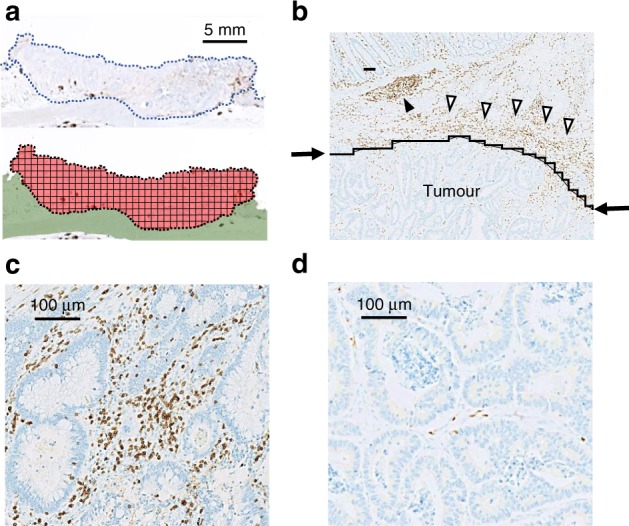
Immunohistochemistry (IHC) of CD3 + tumour-infiltrating lymphocytes (TILs) in colorectal cancer (CRC). **a** Colon tissues were divided into 1-mm^2^ tiles, with tumour tissue highlighted in red. **b** Tumour regions were selected as the area under the curve (indicated by arrows), excluding peritumoural lymphocyte infiltration (open triangle) and extratumoural lymphoid structures (closed triangle). **c** Representative IHC showing high CD3 + cell density. **d** Representative IHC showing low CD3 + cell density

Slides were scanned using a high-resolution digital slide scanner (NanoZoomer-XR C12000; Hamamatsu Photonics, Hamamatsu, Japan), and all tumour lesions were scored automatically by a computerised image analysis system (Tissue Studio; Definiens, Munich, Germany). Measurements were recorded as the mean number of positive cells per tumour tissue unit in square millimetres (Fig. 1a) as well as the number of positive cells among each 1-mm^2^ tissue units. Colorectal cancer tissue may include some lymphoid organs, necrotic tissue or thick fibrous tissue. In this study, only the main cancer lesions which did not include peritumoural lymphocyte infiltration and extratumoural lymphoid structures were selected for analysis (Fig. 1b). The median number of examined sections was one for almost all cases; a maximum of four lesions were examined.

### Analysis of microsatellite instability

DNA was extracted from the resected specimens using the QIAamp DNA FFPE Tissue Kit (Qiagen, Hilden, Germany) according to the manufacturer’s instructions. Microsatellite instability (MSI) status was analysed using the Promega panel of five mononucleotide markers: BAT25, BAT26, NR-21, NR-24 and MONO-27. PCR and subsequent analyses were carried out using the MSI Analysis System **(**Promega, Madison, WI, USA) according to the manufacturer’s instructions. The CRC samples were classified into two groups: MSI-high (MSI-H), when two or more of five markers showed instability; and microsatellite stable (MSS), when zero or one markers showed instability.

### Statistical analysis

Categorical data were assessed using the Chi-square test or Fisher’s exact test. DSS and RFS were defined as the interval from the date of resection to the date of death from cancer and to the date of diagnosis of cancer recurrence, respectively. The survival rate was determined from the date of resection of the CRC until death or until the censor date of 1st May 2017. The distributions of survival time were compared using a log-rank test. Kaplan–Meier curves of DSS and RFS were plotted. Univariate and multivariate analyses were performed using the Cox proportional hazards model. Independent prognostic factors were determined by a stepwise selection process, in which nonsignificant factors were continuously rejected. Statistical analyses were performed using JMP 14 (SAS Institute Inc., Cary, NC, USA). A value of *P* < 0.05 was considered statistically significant.

## Results

### IHC staining and analysis of TILs

**Fig. 2:**
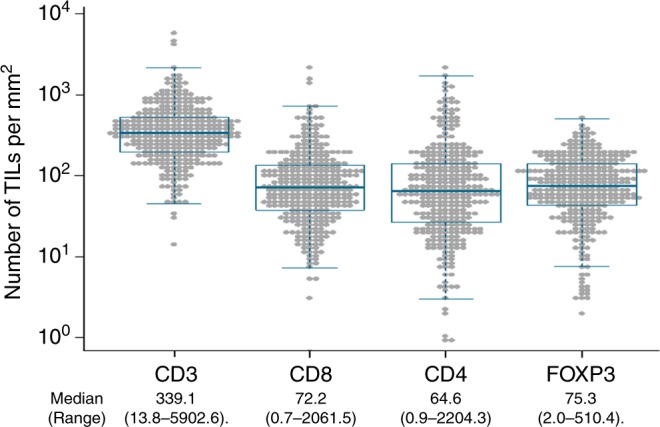
The mean density of tumour-infiltrating lymphocytes (TILs) in colorectal cancer tissues. The number of TILs per mm^2^ was measured by immunohistochemical staining using CD3, CD8, CD4 and FOXP3 antibodies for all patients (342 patients). The median number (cells/mm^2^) and the range are also shown

The IHC staining results of TILs using anti-CD3 antibody are shown in Fig. 1c, d; they were classified into two groups—high- or low-density—according to the median, and representative images show the high- (Fig. 1c) and low-density (Fig. 1d) groups for each TIL. The mean numbers of each TIL per mm^2^ on the IHC-stained cells are shown in Fig. 2.

To clarify the stability of immunostaining in older specimens, the differences in the mean positive cell numbers of all markers between the first-half and second-half of the study period were examined. There were no significant differences between the mean numbers of CD3 + , CD8 +  and CD4 + cells prior to 2001 and after 2002. Although a trend of decreased FOXP3 + cells was observed prior to 2001 compared with after 2002, we determined that these differences would not influence the results of this study (Figure S1).

The number of CD3 + cells among 1-mm^2^ tissue units was evaluated in 15 cases to analyse the intratumoural heterogeneity. Each of five colorectal cancers was selected to this analysis from cases with low, middle and high number of CD3 + cells infiltration. The numbers of CD3 + cells among 1-mm^2^ tissue units were 0 to 205, 0 to 1479 and 0 to 5070 (cells/mm^2^) for low, middle and high infiltration cases, respectively (Figure S2).

### Prognosis according to the density of each TIL

**Fig. 3:**
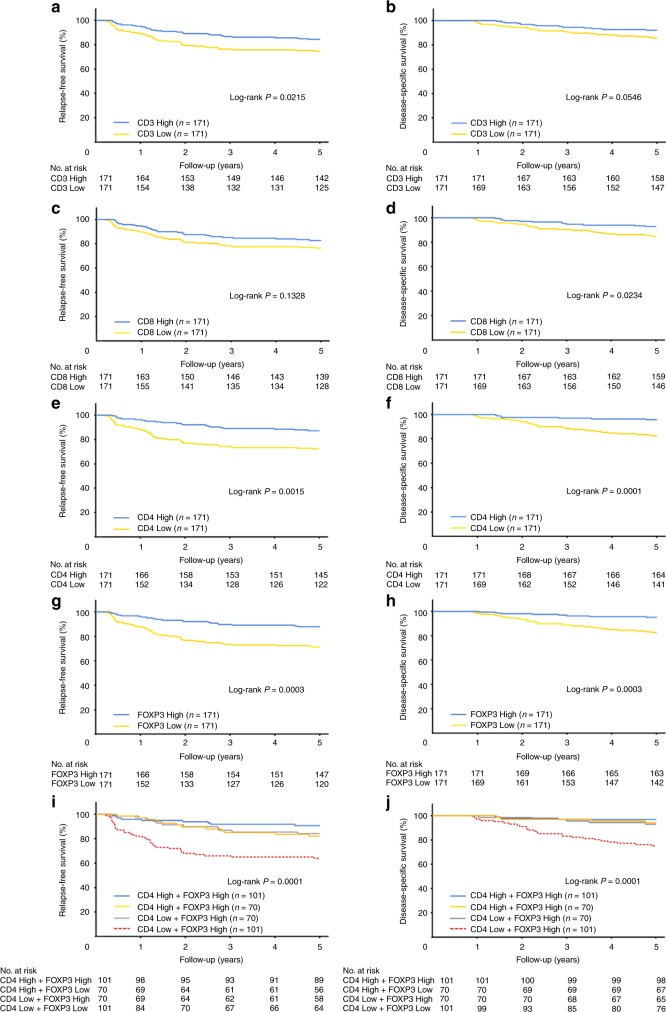
Survival after surgery according to high- and low-density subsets of tumour-infiltrating lymphocytes (TILs). **a** Relapse-free survival (RFS) according to CD3 + cell density. **b** Disease-specific survival (DSS) according to CD3 + cell density. **c** RFS according to CD8 + cell density. **d** DSS according to CD8 + cell density. **e** RFS according to CD4 + cell density. **f** DSS according to CD4 + cell density. **g** RFS according to forkhead box P3 (FOXP3) + cell density. **h** DSS according to FOXP3 + cell density. **i** RFS according to both CD4 + and FOXP3 + cell densities. **j** DSS according to both CD4 + and FOXP3 + cell densities. The cut-off values to distinguish high and low cell density were 339.1, 72.2, 64.6 and 75.3 (cells/mm^2^) for CD3, CD8, CD4 and FOXP3, respectively

We next examined the relationship between the density of each TIL and patient prognosis. In total, there were 37 CRC-specific deaths and 75 recurrences. The Kaplan–Meier survival curves are shown in Fig. 3a–h according to the density of each TIL. Log-rank analysis showed that a high CD8 + cell density (*P* = 0.0234), CD4 + cell density (*P* < 0.0001) and FOXP3 + cell density (*P* = 0.0003) were associated with an improved DSS. Similarly, a high CD3 + cell density (*P* = 0.0215), CD4 + cell density (*P* = 0.0015) and FOXP3 + cell density (*P* = 0.0003) were associated with improved RFS (Fig. 3).

Surprisingly, a combination of CD4 + and FOXP3 + cell density most precisely predicted the prognosis (Fig. 3i, j). Patients with a combination of low CD4 + TIL density and low FOXP3 + TIL density were associated with an extremely poor RFS (*P* < 0.0001) and DSS (*P* < 0.0001) when compared with those with high CD4 + TIL density and low FOXP3 + TIL density, low CD4 + TIL density and high FOXP3 + TIL density, or high CD4 + TIL density and high FOXP3 + TIL density.

### Univariate and multivariate analyses of DSS

Univariate and multivariate analyses of DSS were performed using prognostic factors, such as known clinical factors and MSI status, as well as the density of TILs (Table 2). Univariate analysis revealed that the T stage; histological grade; vascular and lymphatic invasion; adjuvant therapy and the densities of CD8 + , CD4 +  and FOXP3 + TILs were associated with a higher DSS. The MSI status was not shown to be a prognostic factor. In multivariate analyses, the strongest independent prognostic factors were the T stage, vascular and lymphatic invasion, and CD4 + TIL density (Table 3). Moreover, high CD4 + cell infiltration was the strongest factor for a good prognosis (*P* = 0.0004).

**Table 2:** Univariate and multivariate Cox proportional hazard analysis for DSS among patients with CRC

	DSS					
	Univariate			Multivariate		
	HR	95% Cl	*P*	HR	95% Cl	*P*
Sex	1.29	0.68–2.49	0.4363			
Age	1.05	0.54–2.12	0.8866			
Location	0.88	0.46–1.73	0.7118			
T stage	1.89	0.83–4.32	<0.0001	0.72	0.31–1.67	0.0181
N stage	1.82	0.94–3.47	0.0747			
Histologic grade	1.5	0.36–4.19	0.008			
Vascular emboli and lymphatic invasion	0.18	0.07–0.41	<0.0001	0.3	0.12–0.73	0.0029
CD3 density	0.52	0.26–1.01	0.053			
CD4 density	0.22	0.09–0.47	<0.0001	0.27	0.12–0.61	0.0004
CD8 density	0.46	0.22–0.90	0.0221			
FOXP3 density	0.26	0.11–0.54	0.0002			
MSI status	0.81	0.13–2.65	0.7587			
Adjuvant therapy	1.36	0.70–2.75	0.3674			

*DSS* disease-specific survival, *CRC* colorectal cancer, *HR* hazard ratio, *CI* confidence interval, *MSI* microsatellite instability, *MSI-H* microsatellite instability-high, *MSS* microsatellite stable, *FOXP3* forkhead box P3

**Table 3:** Density of TILs in CRC patients according to MSI status (*n* = 322)

	MSI status				
	Patients with MSI-H tumours		Patients with MSS tumours		*P*
	*n* = 23		*n* = 299		
	No.	%	No.	%	
*CD3 density*					0.0212
High	18	78.3	148	49.5	
Low	5	21.7	151	50.5	
*CD8 density*					0.0153
High	17	73.9	144	48.2	
Low	6	26.1	155	51.8	
*CD4 density*					0.8287
High	12	52.2	149	49.8	
Low	11	47.8	150	50.2	
*FOXP3 density*					0.8287
High	11	47.8	150	50.2	
Low	12	52.2	149	49.8	

*TILs* tumour-infiltrating lymphocytes, *CRC* colorectal cancer, *MSI-H* microsatellite instability-high, *MSS* microsatellite stable, *FOXP3* forkhead box P3

### MSI status and T-cell infiltration

MSI status could be measured in 322 cases. The densities of TILs between the MSI-H and MSS groups were compared and are listed in Table 3. The MSI-H group had higher densities of CD3 + and CD8 + cells (*P* = 0.0212 and *P* = 0.0153, respectively) when compared with the MSS group. Interestingly, there was no difference in the distribution of the densities of CD4 + and FOXP3 + cells between the MSI-H and MSS groups (*P* = 0.8287 and *P* = 0.8287, respectively).

## Discussion

In CRC patients, a good prognosis has been reported to be associated with the presence of CD3 + and CD8 + markers of the cytotoxic immune response.^15–20,26^ Consistent with previous reports, this study confirmed the usefulness of CD3 + and CD8 + T-cell densities as prognostic factors (Fig. 3a–d).

The major novel findings of this study were as follows: CD4 + cell density was an independent prognostic factor in our multivariate analysis; FOXP3 + cell infiltration was confirmed to be a positive predictive marker for resectable CRC; the combination of low CD4 + cell infiltration and low FOXP3 + cell infiltration was an accurate prognostic factor for low survival in CRC patients; and the MSI status of tumours was significantly associated with a high CD3 + cell density and CD8 + cell density, but not with a high CD4 + cell density or FOXP3 + cell density.

First, we showed that high CD4 + T-cell density was associated with a positive outcome and was an independent prognostic factor in a multivariate model of CRC; it had greater prognostic value than tumour invasion depth or positive lymph node metastasis (Table 2). To our knowledge, this is the first report to definitively show the usefulness of intratumoural CD4 + T-cell infiltration as a positive prognostic factor in resectable CRC. In our study, we were able to use digitised high-resolution images and specialised software to objectively and quantitatively evaluate the tumour infiltration of immune cells, as we have reported recently;^20,28^ this differed from other studies in which analyses were limited to only parts of tumours selected by the investigators. This analysis of all tumour lesions may help to circumvent observer bias and simplify the measuring technique. Moreover, we selected tumour regions that clearly did not have peritumoural lymphocyte infiltration or extratumoural lymphoid structures (Fig. 1b). Hence, we were able to accurately analyse the intratumoural infiltration of lymphocytes.

CD4 + T-cell density has been reported to be a negative prognostic factor in other types of cancers, e.g. lung, renal, prostate and breast cancer.^28–31^ The reason for this discrepancy remains unclear, but it may be because the function of CD4 + T cells within the tumour microenvironment—i.e. in immune response activation or immunosuppression—may differ depending on the cancer type. Consistent with our results, previous studies have reported that in the absence of regulation by CD4 + cells, specific CD8 + T cells can become lethargic and cannot transform into long-lived functional effector cells,^32,33^ which means that CD4 + T cells have a central role in managing and regulating the immune system against tumour cells. In response to tumours, naive CD4 + T cells can differentiate into four main groups of cells—Th1, Th2, Th17 and T regulatory (Treg) cells—based on cytokine production and regulatory transcription factors.^34,35^ Some studies have revealed that Th1 promotes CD8 + T-cell-mediated adaptive immunity,^36^ that Th1 and cytotoxic gene levels are associated with a good prognosis,^17^ and that CRC patients with high Th17 expression have a poor prognosis. We considered that these complex mechanisms enhanced the immune response in the tumour microenvironment and contributed to the prognosis of CRC.

Next, we confirmed the positive predictive value of FOXP3 + cell infiltration (Fig. 3g, h). CD4 + T cells that express the FOXP3 transcription factor function as Treg cells and suppress effective immune responses against cancer cells.^37,38^ Poor clinical outcomes in various types of cancers are associated with the invasion of abundant FOXP3 + cells into the tumour tissue.^24^ However, contradictory results regarding FOXP3 + cells have been reported in CRC, and cases with high FOXP3 + T-cell infiltration showed a better prognosis in some studies.^25,26,39^ Furthermore, there is substantial evidence that FOXP3 + /CD4 + T cells are functionally and phenotypically heterogeneous, indicating that FOXP3 + /CD4 + T cells can be fractionated based on their expression levels of FOXP3 and CD45RA into FOXP3^low^/CD45RA + naive Treg cells, FOXP3^high^/CD45RA− effector Treg cells and FOXP3^low^/CD45RA− non-suppressive T cells that do not possess suppressive activity and can secrete pro-inflammatory cytokines.^38,40,41^ In the unusual tumour microenvironment of CRC, which contains abundant intestinal bacterial species, non-suppressive FOXP3 + cells were associated with tumour invasion based on the abundance of certain species of intestinal bacteria; *Fusobacterium nucleatum* showed an especially high association with tumour invasion.^42^ Hence, CRC tumours with abundant FOXP3^low^ T-cell infiltration showed a significantly better prognosis than those with high infiltration of FOXP3^high^ T cells.^42^ In our results, a markedly high FOXP3 + cell density was associated with improved prognosis. However, one of the limitations of this study was that the number of FOXP3 + cells was only quantified by immunostaining, and the percentage of non-suppressive T cells remains unknown. Another limitation of this study was that, the numbers of FOXP3 + cells had a trend of decreased prior to 2001 compared with after 2002, and the median number of FOXP3 + T cell in the second-half of the study period was equal to the top 25% in the first period (Figure S1). Hence, the worse survivals in the low-density groups might partially result from the poor prognosis of patients in the old period.

We also found that the combination of CD4 + and FOXP3 + cell densities had the highest predictive value for the prognosis (Fig. 3i, j). This result indicated that the infiltration of only one type of immune cell, i.e. CD4 + or FOXP3 + cells, might be sufficient for a favourable tumour microenvironment to prevent the recurrence of cancer. Although further studies are needed to clarify the scientific mechanism behind these results, our findings may help spark novel ideas and insights on tumour immunity in CRC.

Finally, we found that the densities of CD3 + and CD8 + cells were higher in MSI-H tumours than in MSS tumours, but that the densities of CD4 + and FOXP3 + cells were not affected by the MSI status of the tumour (Table 3). MSI-H tumours, which are caused by a lack of or an alteration in mismatch repair genes, are present in ~6–16% of CRC cases, and are associated with a favourable outcome and a lower potential for metastasis.^43,44^ Our results in CD3 + and CD8 + cells were consistent with those of previous reports, but our results in CD4 + and FOXP3 + cells were not. MSI-H tumours are associated with abundant neoplastic tissue infiltration of CD3 + and CD8 + T cells that can recognise neoantigens.^45,46^ The relationship between CD4 + cells and MSI has not been reported before, and reports on the relationship between FOXP3 + cells and MSI have been contradictory. As in this study, Salama et al. did not observe a significant relationship between FOXP3 + cells and MSI,^25^ and Le Gouvello et al. found a lower mRNA expression level of *FOXP3* in MSI-H tumour tissues.^47^ In CRC, the local infiltration of CD4 + and FOXP3 + cells may be affected by colonic microbiota, rather than by neoantigens. As such, future studies should investigate in detail the correlation between CD4 + T cells and the tumour microenvironment containing colonic microbiota.

In conclusion, we believe that this study is the first to report the prognostic significance of the combination of CD4 + and FOXP3 + T-cell densities detected by IHC in CRC patients. Adjuvant chemotherapy is recommended for stage II and III CRC, and fluoropyrimidines alone or in combination with oxaliplatin regimens are recommended for the appropriate candidates. Therefore, the discovery of these new prognostic indicators is important for the appropriate management of patients undergoing curative resection for CRC. Evaluation of the densities of CD4 + T cells and FOXP3 + T cells may enable the establishment of novel prognostic factors and therapeutic strategies for CRC.

## Supplementary information

Supplementary Information files

Data set

## Data Availability

The datasets are presented in the additional supporting files.
