# Correction: Intratumoural-infiltrating CD4 + and FOXP3 + T cells as strong positive predictive markers for the prognosis of resectable colorectal cancer

**DOI:** 10.1038/s41416-019-0605-4

**Published:** 2019-10-17

**Authors:** Taichi Kuwahara, Shoichi Hazama, Nobuaki Suzuki, Shin Yoshida, Shinobu Tomochika, Yuki Nakagami, Hiroto Matsui, Yoshitaro Shindo, Shinsuke Kanekiyo, Yukio Tokumitsu, Michihisa Iida, Ryouichi Tsunedomi, Shigeru Takeda, Shigefumi Yoshino, Naoko Okayama, Yutaka Suehiro, Takahiro Yamasaki, Tomonobu Fujita, Yutaka Kawakami, Tomio Ueno, Hiroaki Nagano

**Affiliations:** 1 Department of Gastroenterological, Breast and Endocrine Surgery, Yamaguchi University Graduate School of Medicine, Ube, Yamaguchi 755-8505 Japan; 2 Department of Translational Research and Developmental Therapeutics against Cancer, Yamaguchi University Faculty of Medicine, Ube, Yamaguchi 755-8505 Japan; 3 Oncology Center, Yamaguchi University Hospital, Ube, Yamaguchi 755-8505 Japan; 4 Division of Laboratory, Yamaguchi University Hospital, Ube, Yamaguchi 755-8505 Japan; 5 Department of Oncology and Laboratory Medicine, Yamaguchi University Graduate School of Medicine, Ube, Yamaguchi 755-8505 Japan; 6 Division of Cellular Signaling, Institute for Advanced Medical Research, Keio University School of Medicine, Shinjuku, Tokyo, 160-8582 Japan; 7 Department of Digestive Surgery, Kawasaki Medical University, Kurashiki, 701-0192 Okayama Japan

**Keywords:** Colon cancer, Tumour immunology

**Correction to**: *British Journal of Cancer* (2019) **121**, 659–665; https://doi.org/10.1038/s41416-019-0559-6; published online 6 September 2019.

The original version of this article contained an error in Figs. 3i, j. The key for each should read CD4 High + FOXP3 High, CD4 High + FOXP3 Low, CD4 Low + FOXP3 High, CD4 Low + FOXP3 Low. The corrected figure is below. The interpretation of the data and conclusions are not affected.

**Figure:**
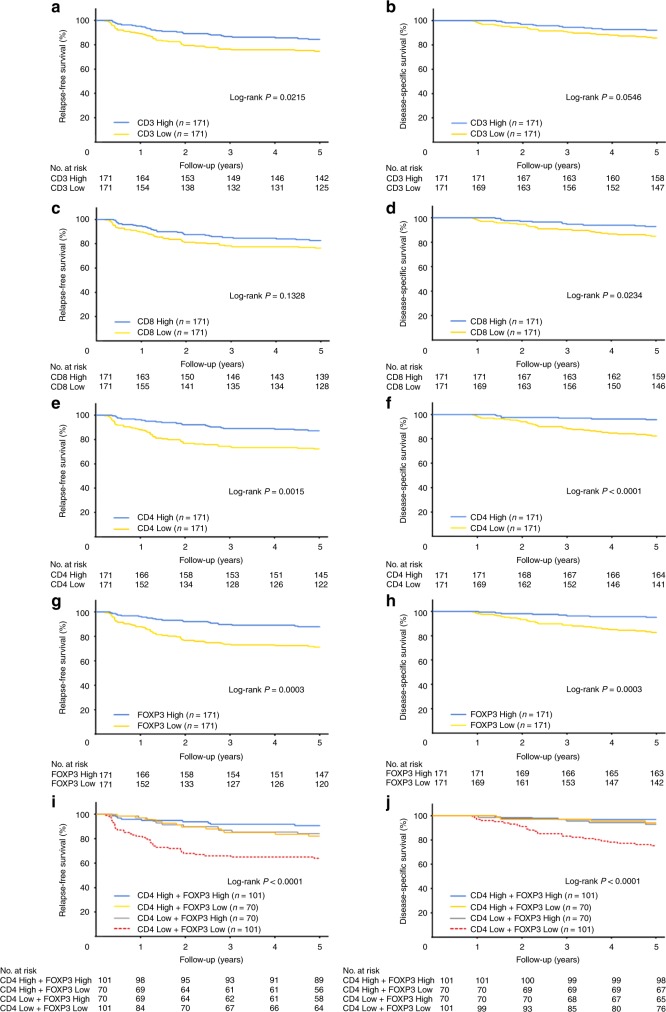
Fig. 3

