# Supplementary material for: Intratumoural-infiltrating CD4 + and FOXP3 + T cells as strong positive predictive markers for the prognosis of resectable colorectal cancer
Source: Br J Cancer. 2019 Sep 6;121(8):659–65. doi: 10.1038/s41416-019-0559-6 (PMC6889292; doi:10.1038/s41416-019-0559-6)
Supplement: Supplementary file 1 — Supplementary Information files [file 41416_2019_559_MOESM1_ESM.pdf]

Table S1. Adjuvant therapy in CRC patients according to disease stage

	Adjuvant therapy: Fluoropyrimidines		
Stage	Yes (n = 198)	No (n = 144)	Total (n = 342)
I	10 (11)	78 (89)	88 (100)
II	91 (64)	51 (36)	142 (100)
III	97 (87)	15 (13)	112 (100)

CRC, colorectal cancer

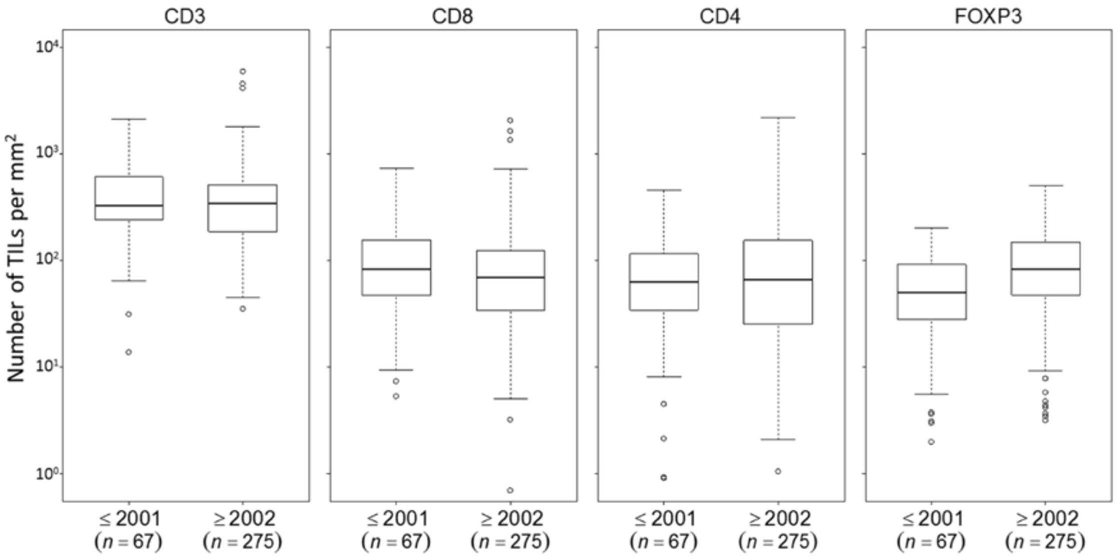

Fig. S1. The positive cell numbers of all markers between the first half and second half of the study period.

There were no significant differences between the mean numbers of CD3+, CD8+, and CD4+ cells among 1 mm^2 tissue units prior to 2001 and after 2002. Although a trend of decreased FOXP3+ cells was observed prior to 2001 compared with after 2002, we determined that these differences would not influence the results of this study.

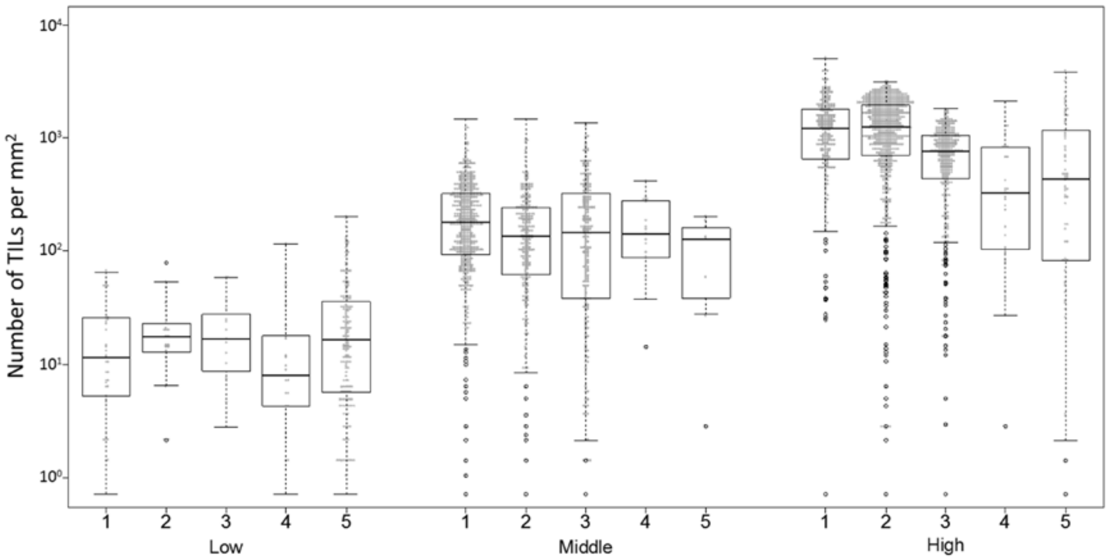

Fig. S2. Distribution of the number of CD3+ cells among 1 mm2 tissue units.

The number of CD3+ cells among 1 mm^2 tissue units was evaluated in 15 cases to analyse the intratumoural heterogeneity. Each of five colorectal cancers was selected to this analysis from cases with low, middle, and high number of CD3+ cells infiltration. The numbers of CD3+ cells among 1 mm^2 tissue units were 0 to 205, 0 to 1479, and 0 to 5070 (cells/mm^2) for low, middle, and high infiltration cases, respectively.
